# Comparison of methods of extracting information for meta-analysis of observational studies in nutritional epidemiology

**DOI:** 10.4178/epih/e2016003

**Published:** 2016-01-11

**Authors:** Jong-Myon Bae

**Affiliations:** Department of Preventive Medicine, Jeju National University School of Medicine, Jeju, Korea

**Keywords:** Meta-analysis, Quality evaluation, Heterogeneity, Nutrition assessment

## Abstract

**OBJECTIVES:**

A common method for conducting a quantitative systematic review (QSR) for observational studies related to nutritional epidemiology is the “highest versus lowest intake” method (HLM), in which only the information concerning the effect size (ES) of the highest category of a food item is collected on the basis of its lowest category. However, in the interval collapsing method (ICM), a method suggested to enable a maximum utilization of all available information, the ES information is collected by collapsing all categories into a single category. This study aimed to compare the ES and summary effect size (SES) between the HLM and ICM.

**METHODS:**

A QSR for evaluating the citrus fruit intake and risk of pancreatic cancer and calculating the SES by using the HLM was selected. The ES and SES were estimated by performing a meta-analysis using the fixed-effect model. The directionality and statistical significance of the ES and SES were used as criteria for determining the concordance between the HLM and ICM outcomes.

**RESULTS:**

No significant differences were observed in the directionality of SES extracted by using the HLM or ICM. The application of the ICM, which uses a broader information base, yielded more-consistent ES and SES, and narrower confidence intervals than the HLM.

**CONCLUSIONS:**

The ICM is advantageous over the HLM owing to its higher statistical accuracy in extracting information for QSR on nutritional epidemiology. The application of the ICM should hence be recommended for future studies.

## INTRODUCTION

A quantitative systematic reviews involving meta-analysis may be applied as an efficient solution to inconsistencies in the outcomes of epidemiological studies [1,2]. However, nutritional epidemiological studies that investigate disease outbreaks caused by food items in regular diet are prone to errors in the course of the meta-analysis of the findings of observational studies such as cohort and case-control studies [3]. Given the problems intrinsic to nutritional epidemiology, such as different research methods, validity of the food frequency questionnaire used, and interregional differences in dietary patterns [4,5], heterogeneity is a factor that should be considered when conducting a meta-analysis of nutritional epidemiological studies [6].

In observational studies related to nutritional epidemiology, dietary intake levels are grouped into 3 to 5 quantiles depending on the predefined categorization, and the effect size (ES) is presented accordingly. As this methodology inevitably poses the problem of inter-study discrepancies in reference points and interval units, only the ES of the highest intake quantile is used for meta-analyses [7]. This ES extraction method, termed the “highest versus lowest” method (HLM), has the following limitations: First, information on the quantiles between the lowest and highest ones are ignored [8]. Second, no clear distinction is made between non-intake and low-intake cases in the lowest-intake quantile [9]. Third, no clear cutoff intake level is set for the highest intake quantile [10].

To overcome these limitations of the HLM, Islami’s collegues presented the interval collapsing method (ICM) [9,11], in which all intervals are taken into account for size calculation. Herein, a meta-analysis is performed by using a fixed-effect model (FEM) to calculate the ES values of all the intervals, which are then collapsed into one ES for the calculation of the summary effect size (SES). This concept is consistent with the method used to calculate the SES after obtaining the collapsed ES through an FEM meta-analysis, with the ES presented according to sex or cancer tissue [12,13]. To investigate the efficiency of applying the ICM to the data of specific food items depending on the exposure source, it is necessary to find out how the ICM outcomes differ from those of the traditional HLM. Therefore, the purpose of this study was to compare the outcomes of the HLM and ICM applied to the same food item in order to determine the advantages and disadvantages of these two methods.

## MATERIALS AND METHODS

The meta-analysis performed by Bae et al. [13] was selected for the outcome comparison between HLM and ICM applications. This article was considered suitable for the purpose of the present study because all 9 observational studies selected for the meta-analysis presented the values in 3 to 5 quantiles of citrus fruit intake levels, and the meta-analysis was performed by extracting the ES of the highest intake group and 95% confidence interval (CI) with respect to the lowest intake group.

Let the reference group be the lowest intake group (*i*=1) and assume that each *k* interval has an odds ratio (OR_i_) and 95% CI, and the ES value obtained by using the HLM is the *k*-layered OR (OR_k_). On the other hand, the ICM was applied after obtaining the ORi and its standard error (SE_i_). The ES values of the respective studies were calculated by using the generic inverse-variance weighted-average method [14]. For example, Stolzenberg-Solomon et al. [15] presented the results on citrus fruit intake of a study in quintiles (Table 1). While the ES of the highest intake quintile (Q5) extracted by using the HLM was 0.79 (95% CI, 0.47 to 1.31), the ES extracted by ICM from the same data was 0.96 (95% CI, 0.75 to 1.22), which was calculated in the FEM meta-analysis based on the ES values of four quintiles (Q2 to Q5).

An FEM meta-analysis was performed on the extraction values obtained from each paper to estimate the SES values and their respective 95% CI obtained when the HLM and ICM were applied. The differences in heterogeneity patterns were tracked by calculating the *I^2^* values. The concordance between the two methods was considered excellent if the SES values maintain the directionality toward null (=1) and no fluctuations in statistical significance occurred based on 95% CI. In addition, the differing patterns were examined by calculating the standard error of log effect size (SElogES) by using the 95% CI of the SES.

## RESULTS

Table 2 lists the ES, 95% CI, and SElogES of the papers selected for the meta-analysis [15-23], arranged to compare the values extracted by using the HLM and ICM. In all 9 articles, no inter-method differences were observed in the directionality of ES and statistical significance, except that the ICM showed a narrower CI and smaller SElogES.

Table 3 was compiled to compare the outcomes of the FEM meta-analysis by using the ES and SElogES values estimated by using the HLM and ICM. The SES and 95% CI showed no inter-method differences in directionality and statistical significance, with SElogSES being smaller in ICM as well. The I-squared values, which are an indicator of heterogeneity, were inconsistent.

## DISCUSSION

Taking these results together, the ICM is advantageous over the HLM in that its outcome values have lower standard errors, hence narrower CIs, while maintaining the directionality and statistical significance of ES and SES.

As a limitation of this study, it should be pointed out that the two methods were comparatively analyzed based on a single meta-analysis. To improve the validity of the conclusions drawn in this study, more validation tests and application examples are required. In particular, as shown in Table 3, while no noticeable differences are observed in the average SES value between HLM and ICM in the 5 cohort studies, the 4 case-control studies show considerable differences between HLM and ICM (0.66 vs. 0.87). Although statistical significance could not be established due to the overlapping 95% CIs, given the remarkable differences in the I-squared values (20.7% vs. 59.6%), further clinical epidemiological research is necessary to determine the magnitude of SES changes depending on the degree of heterogeneity.

Another limitation of this study is the difficulty in interpreting the results obtained by using the ICM, as is the case with the HLM. Islami’s collegues interpreted ICM-estimated ES values as a dietary risk factor for prevalence in comparison with non-intake [9,11], but the reliability of this interpretation should be examined in terms of the methodological aspect. Specifically, dose-response meta-analysis (DRMA) should be performed additionally [24].

In nutritional epidemiology, the application of study results to concrete measures for disease prevention and health promotion projects, and its implementation for the general public can be achieved only when a clear answer can be given to the question of how much of a certain food item should be taken to increase the risk of prevalence. DRMA is currently used, whereby the intake level is converted into a portion size such as daily intake (g/d) [25]. However, DRMA cannot be applied if the related data are presented dichotomously in the selected articles or if intake level cannot be quantified [26]. Keeping in mind a report that 71% of the articles selected for meta-analysis do not lend themselves to DRMA [3], findings from nutritional epidemiological studies should be presented in a manner that would facilitate future meta-analyses. Under the current circumstances, DRMA should be considered as a method to be applied concurrently with the HLM or ICM, instead of replacing them [27-29].

In conclusion, of the methodologies of extracting information for meta-analysis on nutritional epidemiology, the ICM is advantageous over the HLM owing to its higher capacity for statistical accuracy based on a broader information base and should hence be recommended for future research.

## Figures and Tables

**Table 1.** An example of information extraction using the “highest versus lowest” method (HLM) and interval collapsing method (ICM) in the paper by Stolzenberg-Solomon et al. [15]

Citrus fruit intake	Age-adjusted HR (95% Cl)	Extraction	
		by HLM	by ICM
Q5	0.79 (0.47, 1.31)	0.79 (0.47, 1.31)	0.96 (0.75, 1.22)
Q4	1.14 (0.72, 1.81)		
Q3	0.74 (0.44, 1.23)		
Q2	1.15 (0.73, 1.82)		
Q1	1.00 (reference)		

HR, hazard ratio; CI, confidence interval; Q, quintile of intake.

**Table 2.** The effect size (ES) and 95% confidence intervals (CI) obtained by using the two extracting methods: “highest versus lowest intake” method (HLM) and interval collapsing method (ICM)

First author [reference]	Design	HLM		ICM	
		Extracted ES (95% CI)	SE of logES	Estimated ES (95% CI)	SE of logES
Stolzenberg-Solomon [15]	CO	0.79 (0.47, 1.31)	0.2615	0.96 (0.75, 1.22)	0.1236
Coughlin [16]	CO	0.95 (0.82, 1.11)	0.0769	0.94 (0.89, 1.00)	0.0314
Lin [17]	CO	0.95 (0.62, 1.45)	0.2174	0.95 (0.71, 1.27)	0.1470
Larsson [18]	CO	1.12 (0.68, 1.83)	0.2525	1.10 (0.83, 1.45)	0.1439
Nothlings [19]	CO	1.08 (0.82, 1.43)	0.1419	1.04 (0.89, 1.22)	0.0814
Olsen [20]	CC	0.60 (0.30, 1.10)	0.3314	0.89 (0.61, 1.31)	0.1948
Norell [21]	CC	0.44 (0.25, 0.76)	0.2850	0.62 (0.44, 0.89)	0.1835
Ji [22]	CC	0.62 (0.45, 0.87)	0.1704	0.77 (0.64, 0.93)	0.0947
Chan [23]	CC	0.78 (0.58, 1.00)	0.1390	0.85 (0.72, 1.00)	0.0825

SE of logES, standard error of logarithm effect size; CO, cohort studies; CC, case-control studies.

**Table 3.** The summary effect size (SES) and 95% confidence intervals (CI) obtained by using two extracting methods: “highest versus lowest intake” method (HLM) and interval collapsing method (ICM)

Categories	HLM			lCM		
	SES (95% Cl)	SE of logSES	l^2^	SES (95% Cl)	SE of logSES	l^2^
Overall	0.83 (0.70, 0.98)	0.0858	49.9	0.93 (0.88, 0.97)	0.0248	39.5
5 Cohort studies	0.97 (0.86, 1.10)	0.0608	0.0	0.96 (0.91, 1.01)	0.0277	0.0
4 Case-control studies	0.66 (0.55, 0.80)	0.0967	20.7	0.87 (0.80, 0.96)	0.0464	59.6

SE of logES, standard error of logarithm effect size.
