# Supplementary material for: Comparison of methods of extracting information for meta-analysis of observational studies in nutritional epidemiology
Source: Epidemiol Health. 2016 Jan 11;38:e2016003. doi: 10.4178/epih/e2016003 (PMC4751349; doi:10.4178/epih/e2016003)
Supplement: Supplementary file 1 [file epih-38-e2016003-supplementary.pdf]

<Method>

영양역학 관찰연구의 체계적 고찰에서 정보추출 방법 비교

Comparison of Methods of Extracting Information for Meta-analysis of Observational Studies in Nutritional Epidemiology

배종면

Jong-Myon Bae

제주대학교 의학전문대학원 예방의학교실

Department of Preventive Medicine, Jeju National University School of Medicine

Corresponding authors: Jong-Myon Bae

jmbae@jejunu.ac.kr

064-755-5567

Abstract

Objectives: On conducting a quantitative systematic review (QSR) for observational studies related to nutritional epidemiology, only the information in the highest based on the lowest category of a food item (highest versus lowest intake method, HLM) is collected chiefly. Instead of HLM, a method for collapsing categories into a single category (interval collapsing method, ICM) was suggested for using full information of categories. The aim of this study was to compare the effect size (ES) and summary effect size (SES) between HLM and ICM.

Methods: A QSR for evaluating the citrus fruit intake and risk of pancreas cancer calculating the SES by HLM was selected. The ES and SES were estimated by meta-analysis using fixed effect model. The decision of equivalent between HLM and ICM was based on the robustness of direction and statistical significance of ES and SES.

Results: The directions of SES between HLM and ICM were not changed. And ICM showed narrower confidence intervals than HLM

Conclusions: The ICM will be more useful method to extract the related information for QSR on nutritional epidemiology than HLM. It is necessary to promote application of ICM.

Keyword: Meta-analysis, Quality Evaluation, Heterogeneity, Nutrition Assessment

Introduction

역학연구 결과들이 일관성을 보이지 않을 경우 메타분석 (meta-analysis)을 수행한 체계적 고찰 (systematic review) 연구방법론이 유용하다 [1,2]. 그런데, 일상적인 섭취를 하는 식품에 대한 질병발생을 연구하는 영양역학연구에서는 코호트 연구나 환자-대조군연구 같은 관찰연구의 결과에 대한 메타분석을 하는데 이 과정에서 오류가 생길 가능성이 높다 [3]. 연구방법 차이뿐만 아니라 사용한 영양빈도 측정설문도구 (food frequency questionnaire) 타당성 여부, 지역 간 다양한 영양패턴 등 영양역학의 고유한 문제들 [4,5]로 인하여 영양역학의 메타분석에서는 이질성 (heterogeneity)을 반드시 고려해야만 한다 [6].

한편, 영양역학의 관찰연구들은 섭취한 식품에 있어 3-5분위에 따라 구간을 나누고 이에 맞추어 효과크기 (effect size, ES)를 제시한다. 이에 따라 연구마다 구간의 기준점과 단위가 달라지는 문제가 발생하기에, 가장 섭취가 높은 군의 ES만을 메타분석에 적용한다 [7]. 이러한 '최고섭취군' (Highest versus Lowest Method, HLM) 의 ES 추출법은 다음의 한계점들을 가진다. 첫째, 사이 구간의 정보들을 활용하지 못한다는 점이다 [8]. 둘째, 최저 구간이라 해도 경우에 따라서는 전혀 섭취를 안하는 경우와 일부라도 섭취하는 경우가 혼재된다 [9]. 셋째, 최고 구간이라도 섭취량 한계가 불확실하다 [10].

Islami 연구팀 [9,11]은 앞서의 HLM이 가진 한계들을 감안하여 구간을 통합하여 크

기를 산출하는 '구간통합법' (Intervals Collapsing Method, ICM) 을 제안하였다. 이는 고정효과모형 (fixed-effect model, FEM)을 적용한 메타분석을 이용하여 여러 구간의 정보들을 하나의 ES로 산출한 다음, 이를 종합한 Summary Effect Size (SES)를 최종 산출하는 것이다. 이 개념은 성별, 암조직별로 나누어 ES를 제시한 경우, FEM 메타분석을 통해 통합된 ES를 얻어낸 다음 SES 산출에 적용하여 온 것 [12,13]과 일맥상통하다. 그렇지만, 폭로 (Exposure)에 해당하는 특정 식품의 정보들에 있어 ICM법을 적용한다는 것은, 기존의 HLM 법을 적용한 것과는 어떤 차이가 있는지 알아볼 필요가 있다. 따라서 본 연구의 목적은 두 방법 간의 장단점을 알아보기 위하여 HLM과 ICM법을 적용하여 상호 비교하는 것이다.

Materials & Methods

HLM과 ICM을 적용했을 때의 결과를 비교하기 위해서 기존에 발표된 Bae et al. [13] 메타분석 논문을 선정하였다. 선정 이유로는 메타분석을 위해 선정한 9개의 관찰연구 모두 감귤섭취란 항목에 있어 3-5분위 수로 제시한 것이고, 해당 논문은 최저 섭취군을 기준으로 최고섭취군의 ES와 95% 신뢰구간 (confidence intervals, CI)을 추출하여 메타분석을 하였기 때문이다.

한 논문에서 가장 낮은 섭취군 (i=1) 을 기준으로 (reference) 삼은 뒤 k 개의

interval에 따라 OR (OR_i)과 95% CIs 이 있다고 한다면, HLM 법의 ES값은 k 층의 OR (= OR_k) 가 된다. 반면, ICM 법 적용을 위해 OR_i 와 그 표준오차 (standard error) SE_i 를 확보한 다음 generic inverse-variance weighted average 법에 따라 해당 논문의 ES값을 산출하였다 [14]. 이에 대한 예로써 Stolzenberg-Solomon et al. [15] 논문의 감귤섭취 결과를 5분위(quantile)로 제시하였는데 (Table 1), HLM법은 가장 섭취가 높은 Q5의 effect size (ES)인 0.79 (95% CI: 0.47-1.31)를 추출한 반면, ICM은 Q2-Q5 4개의 ES를 FEM에 의한 메타분석으로 산출한 ES 0.96 (95% CI: 0.75-1.22)을 해당 논문의 추출값으로 정하였다.

각 논문별로 확보한 추출 값들에 대하여 FEM 메타분석을 수행하여 HLM과 ICM의 summary effect size (SES)와 그 95% CI를 구하였다. 이질성의 변화양상을 보기 위하여 I-squared 값을 산출하였다. 두 방법 간의 일치성 (equivalent) 여부는 SES 수치가 null (=1)에 대한 방향성이 유지되고, 95% CI에 근거한 통계적 유의성에서 변동이 없는 것을 근거로 판정하였다. 또한, SES의 CI을 이용하여 로그화 표준오차 (standard error of log effect size, selogES)를 산출하여 변동 양상을 살펴보았다.

Results

표 2은 메타분석을 위해 선정된 논문들 [15-23]에 있어, HLM으로 추출했을 때와 ICM으로 산출했을 때의 ES, 95% CI, 그리고 selogES를 비교하기 위하여 정리한 것이다. 9개 모두 두 방법 간에 ES의 방향성과 통계적 유의성에서 변화가 없었다. 다만 ICM의 신뢰구간이 더 좁았으며, SElogES 가 더 작아진 것을 확인할 수 있었다.

표 3는 얻어낸 ES와 SElogES를 이용하여 FEM을 적용한 메타분석 결과를 두 방법간 비교하기 위하여 작성한 것이다. SES 와 95% CI 역시 방향성과 통계적 유의성에서 변화가 없었으며, selogSES 역시 ICM에서 더 작았다. 이질성을 나타내는 I-squared 값은 일관성을 보이지 않았다.

Discussion

이상의 결과들을 요약하자면, HLM 대신 ICM으로 산출한 정보를 활용할 경우, ES 및 SES 의 방향성과 통계적 의미는 유지되면서 표준오차는 작게 되어 신뢰구간을 좁히는 이득을 갖는다.

그러나 단 한 개의 메타분석에 있어 두 방법을 비교검토를 한 것이 본 연구의 한계점이다. 앞서의 결론이 보다 타당하게 되려면 더 많은 확인 작업과 적용 경험이 필요하겠다. 특히 표 3에서 보듯이 HLM과 ICM간의 SBS 크기 변동에 있어 5개의 코호트 연구에서는 변동이 거의 없는 반면, 4개의 환자-대조군 연구에 있어서는 0.66과 0.87로 변동이 있었다. 비록 95% 신뢰구간의 중첩으로 통계적 차이가 없었지만 I-squared 값도 20.7%와 59.6%의 변동을 보였다는 점에서, 향후 이질성 정도의 변화에 따른 SES 변동 크기를 알아

보는 임상역학적 연구가 필요하다.

둘째 한계점은 HLM과 같이 ICM으로 산출한 결과에 대하여도 해석이 여전히 힘들다는 점이다. Islami 연구팀 [9,11]은 ICM을 적용하여 산출된 ES를 해당 음식을 섭취하지 않은 경우에 비하여 섭취했을 때의 발생위험도로 해석하고 있지만, 엄밀히 그렇게 해석할 수 있는가에 대하여는 연구방법론적인 검토가 필요하다. 즉, 양-반응 메타분석 (dose-response meta-analysis, DRMA)를 추가로 적용할 필요가 있다 [24].

영양역학의 경우 연구결과를 질병예방과 건강증진사업에 적용하고 일반인들에게 확산하려면, 해당 식품을 얼마나 섭취해야만 발생위험에 영향을 주는 가에 대하여 답변할 수 있어야 한다. 이를 위해서 일일 섭취량 (gm/day) 같은 portion size로 환산하여 DRMA를 시행한다 [25]. 그러나 선정된 논문에서 관련 정보가 이분형 (dichotomous)으로 제시되거나 섭취량을 양적으로 환산할 수 없을 경우는 DRMA 적용이 불가능하다 [26]. 특히 메타분석을 적용하기 위해 선정한 논문 중 71%는 DRMA를 적용할 수 없다는 보고에 따르면 [3], 식이역학 연구의 결과들은 향후 메타연구에 활용할 수 있도록 제시해야 한다. 따라서 DRMA는 HLM이나 ICM을 대신하기 보다는, 병행하여 적용할 방법론으로 보아야 한다 [27-29].

결론적으로, 영양역학의 메타분석을 위해 추출하는 방법 중 정보를 보다 더 많이 활용하는 ICM이 HLM에 비하여 통계적 정확성의 이득을 더 갖기 때문에, 향후 적극 활성화

할 필요가 있다.

References

1. Morris RD. Meta-analysis in cancer epidemiology. Environ Health Perspect 1994;102 Suppl 8:61-66.

2. Ahn HS, Kim HJ. An introduction to systematic review. J Korean Med Assoc 2014;57:49-59. (Korean)

3. Bekkering GE, Harris RJ, Thomas S, Mayer AM, Beynon R, Ness AR, et al. How much of the data published in observational studies of the association between diet and prostate or bladder cancer is usable for meta-analysis? Am J Epidemiol 2008;167:1017-1026.

4. Gandini S, Merzenich H, Robertson C, Boyle P. Meta-analysis of studies on breast cancer risk and diet: the role of fruit and vegetable consumption and the intake of associated micronutrients. Eur J Cancer 2000;36:636-646.

5. Xu X, Yu E, Liu L, Zhang W, Wei X, Gao X, et al. Dietary intake of vitamins A, C, and E and the risk of colorectal adenoma: a meta-analysis of observational studies. Eur J Cancer Prev 2013;22:529-539.

6. Berlin JA. Invited commentary: benefits of heterogeneity in meta-analysis of data from epidemiologic studies. Am J Epidemiol 1995;142:383-387.

7. Liu XO, Huang YB, Gao Y, Chen C, Yan Y, Dai HJ, et al. Association between dietary factors and breast cancer risk among Chinese females: systematic review and meta-analysis. Asian Pac J Cancer Prev 2014;15:1291-1298.

8. Wu SH, Liu Z. Soy food consumption and lung cancer risk: a meta-analysis using a common measure across studies. Nutr Cancer 2013;65:625-632.

9. Ren JS, Kamangar F, Forman D, Islami F. Pickled food and risk of gastric cancer--a systematic review and meta-analysis of English and Chinese literature. Cancer Epidemiol Biomarkers Prev 2012;21:905-915.

10. Wu W, Kang S, Zhang D. Association of vitamin B6, vitamin B12 and methionine with risk of breast cancer: a dose-response meta-analysis. Br J Cancer 2013;109:1926-1944.

11. Islami F, Ren JS, Taylor PR, Kamangar F. Pickled vegetables and the risk of oesophageal cancer: a meta-analysis. Br J Cancer 2009;101:1641-1647.

12. Bae JM, Lee EJ, Guyatt G. Citrus fruit intake and stomach cancer risk: a quantitative systematic review. Gastric Cancer 2008;11:23-32.

13. Bae JM, Lee EJ, Guyatt G. Citrus fruit intake and pancreatic cancer risk: a quantitative systematic review. Pancreas 2009;38:168-174.

14. Palmer TM, Sterne JAC. Meta-anaylisis in Stata: an updated collection from the Stata Journal. 2nd ed. Texas: Stata Press Publication; 2016, p. 25.

15. Stolzenberg-Solomon RZ, Pietinen P, Taylor PR, Virtamo J, Albanes D. Prospective study of diet and pancreatic cancer in male smokers. Am J Epidemiol 2002;155:783-792.

16. Coughlin SS, Calle EE, Patel AV, Thun MJ. Predictors of pancreatic cancer mortality among a large cohort of United States adults. Cancer Causes Control 2000;11:915-923.

17. Lin Y, Kikuchi S, Tamakoshi A, Yagyu K, Obata Y, Inaba Y, et al. Dietary habits and pancreatic cancer risk in a cohort of middle-aged and elderly Japanese. Nutr Cancer 2006;56:40-49.

18. Larsson SC, Håkansson N, Näslund I, Bergkvist L, Wolk A. Fruit and vegetable consumption in relation to pancreatic cancer risk: a prospective study. Cancer Epidemiol Biomarkers Prev 2006;15:301-305.

19. Nöthlings U, Murphy SP, Wilkens LR, Henderson BE, Kolonel LN. Dietary glycemic load, added sugars, and carbohydrates as risk factors for pancreatic cancer: the Multiethnic Cohort Study. Am J Clin Nutr 2007;86:1495-1501.

20. Olsen GW, Mandel JS, Gibson RW, Wattenberg LW, Schuman LM. Nutrients and pancreatic cancer: a population-based case-control study. Cancer Causes Control 1991;2:291-297.

21. Norell SE, Ahlbom A, Erwald R, Jacobson G, Lindberg-Navier I, Olin R, et al. Diet and pancreatic cancer: a case-control study. Am J Epidemiol 1986;124:894-902.

22. Ji BT, Chow WH, Gridley G, Mclaughlin JK, Dai Q, Wacholder S, et al. Dietary factors and the risk of pancreatic cancer: a case-control study in Shanghai China. Cancer Epidemiol Biomarkers Prev 1995;4:885-893.

23. Chan JM, Wang F, Holly EA. Vegetable and fruit intake and pancreatic cancer in a population-based case-control study in the San Francisco bay area. Cancer Epidemiol Biomarkers Prev 2005;14:2093-2097.

24. Greenland S, Longnecker MP. Methods for trend estimation from summarized dose-response data, with applications to meta-analysis. Am J Epidemiol 1992;135:1301-1309.

25. Berlin JA, Longnecker MP, Greenland S. Meta-analysis of epidemiologic dose-response data. Epidemiology 1993;4:218-228.

26. Aune D, Chan DS, Vieira AR, Navarro Rosenblatt DA, Vieira R, Greenwood DC, et al. Red and processed meat intake and risk of colorectal adenomas: a systematic review and meta-analysis of epidemiological studies. Cancer Causes Control 2013;24:611-627.

27. Han J, Jiang Y, Liu X, Meng Q, Xi Q, Zhuang Q, et al. Dietary Fat Intake and Risk of Gastric Cancer: A Meta-Analysis of Observational Studies. PLoS One 2015;10:e0138580.

28. Vieira AR, Abar L, Vingeliene S, Chan DS, Aune D, Navarro-Rosenblatt D, et al. Fruits, vegetables and lung cancer risk: a systematic review and meta-analysis. Ann Oncol 2015. pii: mdv381. [PMID: 26371287]

29. Vieira AR, Vingeliene S, Chan DS, Aune D, Abar L, Navarro Rosenblatt D, et al. Fruits, vegetables, and bladder cancer risk: a systematic review and meta-analysis. Cancer Med 2015;4:136-146.

Conflict of Interest

The author has no conflict of interest to declare for this study.

국문요약

영양역학과 관련하여 관찰연구 결과를 체계적 고찰을 할 때, 통상적으로 섭취가 가장 낮은 군을 기준으로 하여 가장 높은 군의 effect size (ES) 정보를 추출하는 Highest versus Lowest Method (HLM)을 적용한다. 그러나 주어진 정보를 최대한 활용한다는 점에서 구간의 정보들을 통합한 ES 정보로 메타분석에 적용하는 Interval Collapse Method (ICM)법이 제안되었다. HLM에 비하여 더 많은 정보를 활용한 ICM 적용을 한 결과, ES 뿐만 아니라 SES에서도 일치성을 보이면서 표준오차가 작아지는 현상을 확인하였다. 영양역학의 메타분석을 위해 추출하는 방법 중 정보를 보다 더 많이 활용하는 ICM이 HLM에 비하여 통계적 정확성의 이득을 더 갖기 때문에, 향후 적극 활성화할 필요가 있다.

ORCID : 0000-0003-3080-7852
